# Perceived barriers and motivating factors influencing student midwives’ acceptance of rural postings in Ghana

**DOI:** 10.1186/1478-4491-10-17

**Published:** 2012-07-24

**Authors:** Jody R Lori, Sarah D Rominski, Mawuli Gyakobo, Eunice W Muriu, Nakua E Kweku, Peter Agyei-Baffour

**Affiliations:** 1 School of Nursing, University of Michigan, Ann Arbor, MI, 48109, USA; 2 Global REACH, University of Michigan Medical School, Ann Arbor, MI, 48109, USA; 3 University of Ghana School of Medicine, Accra, Ghana; 4 University of Michigan, Ann Arbor, MI, 48109, USA; 5 School of Medical Sciences, Kwame Nkrumah University of Science and Technology, Kumasi, Ghana

**Keywords:** Africa, West, Health care, Human resources for health, Maternal health, Midwifery, Qualitative Methods, Recruitment, Retention, Rural

## Abstract

**Background:**

Research on the mal-distribution of health care workers has focused mainly on physicians and nurses. To meet the Millennium Development Goal Five and the reproductive needs of all women, it is predicted that an additional 334,000 midwives are needed. Despite the on-going efforts to increase this cadre of health workers there are still glaring gaps and inequities in distribution. The objectives of this study are to determine the perceived barriers and motivators influencing final year midwifery students’ acceptance of rural postings in Ghana, West Africa.

**Methods:**

An exploratory qualitative study using focus group interviews as the data collection strategy was conducted in two of the largest midwifery training schools in Ghana**.** All final year midwifery students from the two training schools were invited to participate in the focus groups. A purposive sample of 49 final year midwifery students participated in 6 focus groups. All students were women. Average age was 23.2 years. Glaser’s constant comparative method of analysis was used to identify patterns or themes from the data.

**Results:**

Three themes were identified through a broad inductive process: 1) social amenities; 2) professional life; and 3) further education/career advancement. Together they create the overarching theme, *quality of life*, we use to describe the influences on midwifery students’ decision to accept a rural posting following graduation.

**Conclusions:**

In countries where there are too few health workers, deployment of midwives to rural postings is a continuing challenge. Until more midwives are attracted to work in rural, remote areas health inequities will exist and the targeted reduction for maternal mortality will remain elusive.

## Introduction

Midwives are the foundation of maternal and child health worldwide. Their knowledge and skills have been identified as necessary to achieve the Millennium Development Goal (MDG) Five, to improve the condition of mothers giving birth and to reduce by 75% the number of women who die in childbirth by 2015 [1]. The World Health Organization (WHO) [2] has identified a need for 334,000 additional midwives in the world to meet the reproductive needs of all women. To meet this demand, midwifery education programs are growing exponentially in some countries, increasing the workforce by hundreds, and in some cases thousands, of midwives in a country each year. Despite the on-going efforts to increase this cadre of health workers there are still glaring gaps and inequities in distribution.

## Background

Uneven distribution of health care workers contributes to the continued inequity in health outcomes [3]. The staffing of public sector health facilities in remote rural areas is a serious challenge for many ministries of health in low- and middle-income countries. One of the biggest challenges of under-resourced health systems is the difficulty they face in producing, recruiting, and retaining health professionals, particularly in remote areas [4]. Confronting geographical imbalances in the workforce continues to test health systems and contributes to the lack of progress in achieving MDG Five.

In a study of 31 sub-Saharan African countries, attendance by a skilled health care provider at birth between 1990 and 2000 remained stagnant with less than 40% of births attended by a doctor, nurse or midwife [5]. This shortage often impacts heavily on the poorest people in the least developed regions of the world [6]. Health care systems face numerous difficulties in adequately staffing rural, remote areas.

The mal-distribution of the health care workforce has been widely documented [7-10] but focuses mainly on physicians and nurses. According to the WHO [11] the shortage of qualified health workers in rural areas – where half of the world’s population lives – significantly impacts the attainment of the MDGs. A clear understanding of the specific situation, cultural context and needs of a country and its workforce are advocated if we are to make an impact on the development of programs and policies to attract workers to rural, remote areas [11].

### Ghana

Overall, the WHO African Region, which carries 24% of the global burden of disease, is served by only 3% of the world’s health care workers [12]. The shortage of qualified health care workers in rural areas of Ghana continues to be an obstacle to achievement of MDG Five.

Maternal death in Ghana is currently estimated at 350 per 100 000, in part a reflection of the low rates of skilled support during birth [13]. According to the Demographic Health Survey, 43% of Ghanaian women give birth alone or with a non-skilled attendant [14] although 62.8% of women surveyed had attended the minimum standard of four antenatal visits [15]. A breakdown of maternal mortality by region indicates pregnant women have unequal chances of survival across the country, with mothers in the Greater Accra area experiencing the highest chances of survival [16].

Like most sub-Saharan African countries, Ghana is experiencing a critical shortage of midwives. In 2000, the exodus of more than 500 nurses and midwives from Ghana to industrialized countries accounted for more than twice the number of new nurses and midwives graduating from programs in the country that year [17]. In 2006 there were approximately 17 322 nurses and midwives working in Ghana, while 2267 migrated to developed countries. This figure represents 13% of the total nurse and midwife workforce, placing Ghana fourth in the rankings of sub-Saharan countries suffering from nurse and midwife migration [18]. In 2011, there were 3780 midwives practicing in Ghana, representing 5 midwives per 1000 live births or 84 midwives per 100 000 population. Midwives attend approximately 65% of all births in urban settings and 35% of births in rural areas [13].

An aging midwifery population also contributes to the diminishing cadre of practicing midwives in Ghana. In a survey of 145 midwives [18], 79% of those surveyed were between 41 and 60 years old (39% between ages 41 and 50 and 40% between ages 51 and 60), 10% were between 20 and 40 years, and 6% between 61 and 80 years of age. Of the midwives surveyed, 5% did not report their age. With mandatory retirement at age 60 in Ghana, the pool of practicing midwives will soon diminish. There are currently 14 midwifery training colleges in Ghana, with at least 1 school in each of the country’s 10 regions, graduating 500 to 600 midwives per year [19].

A literature review examining staffing in remote, rural areas in middle- and low-income countries found that 1247 (87.2%) general physicians in Ghana worked in urban regions although 66% of the population resides in rural areas [4]. The Ghana Ministry of Health employs a number of incentives to recruit and retain health staff to rural, deprived areas. These include a 20% to 30% salary top up for health staff in deprived areas implemented from 2004 and a staff vehicle purchase scheme since 1997 [20].

There is currently very little data examining the motivation for midwives to accept rural postings after graduation. This paper examines perceived barriers and motivating factors that influence student midwives’ willingness to work in remote, rural areas of Ghana.

## Methods

### Study settings and participants

Our study was conducted in 2009 in Accra, the capital city, and Kumasi, the second largest city in Ghana, as part of a larger study to further the understanding of factors that motivate health care professionals to move to rural areas. These two cities are the main economic “nerve center” of Ghana and retain most of the health professionals in the country.

Final year midwifery students, waiting to write their professional examinations, from the two largest midwifery training schools, Korle Bu and Komfo Anokye Teaching Hospitals, were purposively selected for the study. The use of purposive sampling ensured diversity of participants’ views, opinions and experiences in rural areas. Moreover, the two training schools chosen form the core of professional midwives in Ghana. These students’ views demonstrate a broad perspective.

A descriptive study design was used to explore the barriers and motivating factors that could influence final year midwifery students to accept a job posting to a rural, deprived area. Focus group interviews were selected as the data collection strategy to allow participants to reflect on their own experiences as well as provide an opportunity to hear others tell of their experiences and build on those viewpoints [21]. Focus group interviews afford the opportunity for participants to provide contextual meanings and insights as well as to validate findings that are less accessible without the interaction found in a group setting [22].

We obtained ethical approval from the Ghana Health Service Ethical Review Committee, the University of Ghana Medical School, the Kwame Nkrumah University of Science and Technology Committee on Human Research, Publications and Ethics, and the University of Michigan Institutional Review Board.

### Study design and data collection

This exploratory qualitative study used focus group discussions to collect data. All final year midwifery students were invited to participate in the focus groups. Of a total of 225 final year midwifery students at both schools, 49 participated in 6 focus groups. Four focus group discussions were held at the school with the larger class size (148 students) and two focus groups were held at the second school with a class size of 77 students. To assist with recruitment and minimize faculty burden, flyers were posted around the schools with the place and time for the focus group discussions. Students were told they would receive an incentive following the interviews in the form of a phone card worth GH¢7.00 (approximately 5 US dollars).

Interview guides were developed collaboratively with United States of America and Ghanaian partners with the following aims: 1) to elucidate students’ perceptions of rural, deprived areas; 2) to identify students’ plans for employment following graduation; 3) to capture students’ experiences of working or living in rural areas; and 4) to describe students’ preferences for rural versus urban practice for future employment.

Prior to data collection informed consent was obtained from participants. Participants were informed interviews would be tape recorded and that no identifying information would be used. Six focus groups were held in a private space within the two schools of midwifery. Group size varied from 7 to 12 participants. Data collection continued until saturation was achieved and no new information was being uncovered. The focus group sessions lasted 60 to 90 minutes and were led by a team of one Ghanaian and one United States investigator. One investigator facilitated the focus group and the second investigator took notes and monitored the recording equipment.

After an introduction to the study by the researchers, brief opening questions related to previous experience in rural, deprived areas and plans after graduation from midwifery school were discussed. Students were then presented with the following definition from the ministry of health of a deprived area: “An area distant from the big cities and lacking social amenities like schools, roads, pipe borne water*.”* Focus group questions and probes included: “What are your top preferences for places to work after graduation?” “How likely are you to consider working in a deprived area?” “What are the main barriers for accepting a posting to a deprived area?” “What are the main motivators for accepting a position to a deprived area?*”*

### Analysis

Analysis was guided by the research question “What barriers and motivating factors influence student midwives’ acceptance of rural postings following graduation?*”* All data were transcribed verbatim by local research assistants and transcripts were uploaded to Nvivo 9 software for analysis. Glaser’s constant comparative method of analysis [23,24] was used to identify themes related to perceived barriers and motivating factors for working in a deprived area following graduation.

### Rigor

The primary investigator and two additional members of the research team analyzed the data separately and then together at team meetings until common themes emerged. First, the transcripts were read and reread line-by-line and open coding was conducted to identify incentives and conditions that would make working in a deprived area attractive to participants. Over the six focus groups these codes were compared then assigned to categories that appeared to cluster together and finally into common themes to explain the data. Data were discussed until agreement was reached and an audit trail was maintained to document decisions throughout the process. Additionally, an environment of reflexive self-examination of our own biases and assumptions was upheld as a continual effort to ensure trustworthiness.

### Limitations

This study was conducted at schools located in two of the most urban settings in Ghana. It is unknown whether students from other Ghanaian midwifery schools, particularly those located in a more rural setting perceive the barriers and motivators for rural practice in the same way as those represented in this study.

## Results

The participants in our study (n = 49) were all female, young (average age of 23.2 years) and all but one did not have children. The overarching theme revealed by the data analysis was *quality of life*. Although participants voiced a strong desire to help others and to save lives, they also expressed a deep concern for their own quality of life and how living in a rural, deprived area would impact them. The three themes we identified through a broad inductive process included 1) social amenities; 2) professional life; and 3) further education/career advancement. Together they create the overarching theme, *quality of life*, that we use to describe the influences on midwifery students’ decision to accept a rural posting following graduation.

### Social amenities

Many of the students noted aspects of their personal life that might preclude them from accepting positions in a rural area. These included unacceptable housing accommodations, no access to potable water, impassable roads, no access to entertainment, no transportation, poor or nonexistent schools for future children, insufficient lighting and local people who are ignorant and, therefore, do not make ideal patients and would not make suitable husbands. The term “*social amenities*” was consistently used by the participants and had not been heard by the study team prior to these focus groups. As one participant noted,” I think when we talked about deprived areas, they are areas that lack social amenities or areas with inadequate social amenities for good living.’”

The idea of living in an area that lacks what is necessary to live a good life arose multiple times. The vast majority of young women in our sample was single and anticipated looking for a partner in the future. Many expressed the desire for children in the future. For them, the prospect of meeting someone in an urban area was much greater. As one participant said:

"*“*I am still single and I am still searching for [a partner]. So I am not able to go to the rural areas and find my [husband], but I have to be here and find someone and have a family before maybe I can do something else [like work in a rural, deprived area].”"

Participants shared concerns about their physical comfort, and being surrounded by family and those who they identify as like them and important to their personal well-being. Relocating to an area that lacks basic necessities such as water and lighting is something they have little interest in.

"*“*There should be schools; there should be good drinking water. There should also be good lighting system. Also the people [in rural areas] should enjoy everything that the people in the city are enjoying so that my work wouldn’t be a burden for me. When they are enlightened it makes your work easier. When you are giving health education they understand what you are doing. So that’s what I want the government to do.”"

As part of the midwifery education and training, students experience working in deprived areas during clinical rotations to district hospitals and rural clinics. Participants reported these experiences as being nearly unbearable. One young woman recalled:

"“I lived in a deprived area near Accra. We have a poor drinking system and then people dispose [waste] around. And also our roads are very poor. Also they switched off the light anytime they want. Also the water system will leave up to 2 months [and no] water will be flowing. They won’t give you notice or anything, so it is very bad*.*”"

Students in our focus groups also wanted to offer educational opportunities to their future children. They view deprived areas as having poor educational facilities for children*.*

"*“*I cannot afford to marry someone from the rural area as well raise my children in the rural areas because they will not have good schools to attend. I can’t raise my family in the rural area. Never. I want them to attend a good school so that they will have a vying chance and all that.”"

Finally, one young woman said:

"“So the kind of life that I want to live; the rural areas will not permit me to get that kind of life. And my children won’t get the best of education. So I would like to work in the urban areas rather so that I’ll get all that I want in life. That’s what I think. So if the government will be able to give me all the things that I was having in the urban areas I definitely may choose the rural areas.”"

### Professional life

The students in our sample mentioned issues regarding their future professional lives often. They believed deprived areas would not offer the type of support structures within a facility required to practice in the manner they were taught at school. They stressed the importance of an environment conducive to using their newly learnt skills in caring for childbearing women. Most believed they would have to refer women to better equipped facilities.

“And they don’t have enough facilities [in deprived areas]. In this care they will refer, refer, refer. So when you go to such places you do not get a chance to observe or get those interesting cases. They will refer to well facilitated [equipped] hospitals. So when you are posted over there you are outmoded.”

Participants stated midwives currently in the rural areas are all “older and have not been in school for many years*”.* Due to age and seniority, these midwives would be their supervisors. Students were concerned they would not be able to practice in the way they were taught because their supervisors would not know the new methods they had been taught. As expressed by one young woman:

“In the rural areas there are no kind of workshops organized for the midwives over there so it seems that everything that we are taught here - new deliveries and anything that we are taught in the classrooms here - is different than what is being practiced over there. For instance when you go for the district [clinical immersion] the midwife is conducting the delivery everything is wrong when you compare it to what you were taught in the class. When you try to convince the midwife to use the method that you were taught, it will be some kind of you telling the midwife what to do. You saying what she is doing is wrong. So when I complete my training and I am sent there I will be compelled to work like they do because they think what they are doing is best. I would also not like to work like that because the implications and consequences of some of the actions that they do.”

Participants in our sample believed midwives in urban areas had greater access to skills workshops and continuing education than those posted in a rural area. For example one participant said*:* “[There is] a lack of in-service training; [for] example when you’re in the training and there are new issues coming up, your ability to get access to such information is limited.”

Another student said:

"“In the rural area there is a lack of universities that one can upgrade or continue with education. And then there is the abandonment by the government; you are left there…till you go on pension.”"

Some of the young women in our study also mentioned the lack of staff in the rural areas. They discussed being stuck in rural areas, unable to return to the urban areas to continue their education. They also believed that in rural areas you are expected to work 24 hours a day and never have any time for yourself.

"“In rural areas they lack staff. And so if you are young and you go there and you feel like you want to go back to school they wouldn’t release you. They would just say please stay because we lack people to work. And yes, they wouldn’t allow you to go to school."

"So when you are there you are solely responsible for all…twenty four hours you’ll be there.”"

Further, these students voiced the concern that once they accept a posting to a deprived area, they will be stuck there indefinitely. They do not believe they will have access to return to urban areas but will be expected to live their entire lives in the deprived areas. One of the participants said:

"“It’s difficult normally. They will tell you there is a shortage of staff and is difficult to be released and I am young so I think I will rather would like to start from the urban and later on move to the district, yes!”"

### Further education/career advancement

Following closely, and often overlapping with, the theme of professional life is the theme of further education and career advancement. A large number of our participants noted that, following the completion of their midwifery diploma, they would like to, in their words, “further my education”*.* This meant different things to many of them. Some students mentioned obtaining a degree in midwifery, a new opportunity beginning for the first time shortly after these data were collected. Others mentioned getting degrees in nursing or administration. Some of our participants wanted to enter medical school. Some wanted to become midwifery tutors. Although their future desires differed, what remained consistent throughout the data was that very few felt they were finished seeking formal education. Although the ministry of health is hoping many of these young women will begin practicing as midwives following their graduation from the newly created diploma programs, it was clear many had other ideas. As one young woman said:

"“It’s like when I get there [a deprived area] that means I cannot come back to continue my education. Like say taking me to a village in the Western region and bringing me back to maybe Cape Coast University is very far.”"

Many of our participants recognized that women in the deprived areas need help during childbirth. Although they were cognizant of the need, they continued to voice their own reasons for not wanting to move to a deprived area. For example:

"“I would love to work in the district but because of the situations in the country currently [it] is difficult. So I will rather prefer the urban area so that I will be able to go to school easily.”"

Some of the participants mentioned connecting further education to rural service would be a way to incentivize them to practice in these areas.

"“And moreover like, sponsorship to further education, what we’ve realized is those who go to the rural areas they lack further education. Always those in the urban areas will have access to further their education. So it’s like, if there is any information, you just imagine even the Dailies (newspapers). It’s very difficult for the dailies to get to the rural areas. So that if you are there how will you read the dailies to find out that there’s an opportunity for you to further your education there? So if there are incentives, like if you are in the rural areas after three years you will be given a chance to go and further your education and come back to work there, I would like to go there.”"

## Discussion

Access to skilled birth attendants and healthcare workers is negatively associated with both maternal and infant mortality. For many people living in rural Ghana, there is no available skilled birth attendant within a reasonable distance [14]. Although these soon-to-be-graduating midwives fully understand the problems facing rural women, only two of the students said that they were likely to locate to rural areas to practice midwifery after graduation.

Our findings corroborate earlier research on the barriers and motivators for rural practice among other health professionals such as physicians and nurses [25-27]. Although many young women in our sample mentioned altruistic reasons for going into midwifery, their own personal quality of life, including social amenities, professional practice and a desire to further their education made location to rural areas unappealing. In the local Twi dialect students’ often said: “dabi, dabi” which translates into “no way”.

Quality of life, including access to the basic social amenities of clean water, adequate housing, and schools for children, was strongly voiced as a barrier to remote postings. Strategies which address these issues would benefit not only the health sector but the entire population [4]. A supportive working environment and co-workers were cited as important to our participants. Rural workers often need a deeper and wider set of skills to be able to deal with complex health problems that in urban areas are managed by a team of healthcare providers [6].

Our findings support the WHO [6] recommendations to develop and support career development programs for workers in rural areas. The opportunity for career advancement was a major consideration for the midwifery students participating in our focus groups.

Considering the demographics of our sample as representative of the larger student body, attention to our findings and the findings of the computer based survey which followed should be used to inform public policies. Nearly all of the participants in the computer-based survey [28] reported they plan to have children in the future; issues around providing for future children are highly important to them.

The participants in these focus groups did seem willing to entertain the notion of re-locating to rural areas for a short, defined period of time, especially if this service could increase their chances of furthering their education upon their return. Although material incentives, such as housing and increased salary, were mentioned, the desire that these young women feel for obtaining more education could be a relatively inexpensive policy initiative to attract young midwives into the rural areas for fixed amounts of time. Further, working to improve the skills of all practicing midwives may make rural facilities more attractive.

## Conclusions

The current body of evidence suggests that incentives to redistribute the health care workforce in sub-Saharan Africa have not worked to date. This uneven distribution extends to midwifery professionals as well.

New recommendations from the WHO based on a systematic analysis of the literature on education, recruitment, and retention of rural health care workers, reflect the personal and professional support as well as education recommendations themes found in our study. Some of these include: continuing education and professional development programs accessible from rural and remote locations, improved living conditions for health workers and their families, supporting the development of professional networks, and providing appropriate equipment and supplies as well as supportive supervision and mentoring for new recruits to rural areas [6].

In many countries midwives are the main providers of obstetrical care. Midwives have the greatest potential to improve the reproductive health of women and save the lives of mothers and infants in many parts of the developing world [29]. In countries where there are too few health workers, deployment to rural postings is a continuing challenge. Until more midwives are attracted to work in rural, remote areas health inequities will exist and the targeted reduction in maternal mortality will remain elusive.

## Competing interests

The authors declare that they have no competing interests.

## Authors’ contributions

All authors contributed to all aspects of the research study described above. Specifically, JL made substantial contributions to conception and design of this study. JL also spent significant time revising the manuscript for important intellectual content as well as giving final approval for the version to be published. SR worked with JL to conceptualize the study as well as collecting, analyzing and interpreting the data. SDR also spent considerable time drafting and revising the manuscript. MG was heavily involved in the collection of data as well as the revising of the manuscript. EM was involved in data collection as well as initial drafting of the manuscript. NK was involved in data collection and manuscript revision. PA-B was involved in data collection and manuscript revision as well as approval of the final version to be published. All authors read and approved the final manuscript.
